# Laparoendoscopic extraperitoneal surgical techniques for ventral hernias and diastasis recti repair: a systematic review

**DOI:** 10.1007/s10029-024-03144-3

**Published:** 2024-09-23

**Authors:** Francesco Ferrara, Federico Fiori

**Affiliations:** 1 Department of Precision Medicine in Medical, Surgical and Critical Care (Me.Pre.C.C.), University of Palermo, Palermo, Italy; 2 Department of Surgery, Unit of General and Oncologic Surgery, “Paolo Giaccone” University Hospital, Palermo, Italy; 3 Department of Emergency, Unit of General and Emergency Surgery, “San Carlo Borromeo” Hospital, ASST Santi Paolo e Carlo, Milan, Italy

**Keywords:** Laparoscopy, Endoscopy, Abdominal wall surgery, Hernia, Diastasis recti

## Abstract

**Purpose:**

this systematic review aims to classify and summarize the characteristics and outcomes of the different laparoendoscopic extraperitoneal approaches for the repair of ventral hernias and diastasis recti described in the last 10 years.

**Methods:**

a literature search was performed by two reviewers in December 2023 including articles from January 2013, 01 to December 2023, 15. The techniques were selected according to the surgical access site (anterior or posterior to the rectus sheath), the access type (laparoendoscopic, single incision laparoscopic, mini or less open), the main space used to repair the defect (subcutaneous or retromuscular) and the mesh place (onlay, sublay-retromuscular or sublay-preperitoneal) and classified as anterior or posterior approaches.

**Results:**

the literature search retrieved 1755 results and 27 articles were included in the study. The studies included 1874 patients, the mean age ranged from 37.8 to 60.2 years. The access site was anterior in 16 cases and posterior in 11 cases. The mesh was positioned onlay in 13 cases and sublay in 13 cases, with only one study using no mesh. Complications were: seroma, ranging from 0.8 to 81%, followed by skin complications (leak, ischemia, necrosis) from 0.8 to 6.4%, surgical site infections and bleeding. Recurrences ranged from 0% to 12,5%, with a mean follow-up from 1 to 24 months.

**Conclusion:**

this systematic review confirms the presence of several new minimally invasive extraperitoneal techniques for the repair of abdominal wall defects, with different advantages and disadvantages. Further studies, with more extensive follow-up data and wider patient groups, are necessary to define specific indications for each technique.

## Introduction

Abdominal wall defects, including ventral hernias and other structural anomalies like diastasis recti (DR), pose significant challenges to both patients and surgeons [1]. The field of minimally invasive surgery, including laparoscopic, robotic and endoscopic techniques, has witnessed remarkable progress over the past few decades, revolutionizing the approach to abdominal wall defects [2, 3], leading to improved cosmetic outcomes and reduced risk of chronic pain [4]. Among these, the Intraperitoneal Onlay Mesh (IPOM), described in 1993 by Le Blanc et al., has emerged as a pioneering approach, representing a significant paradigm shift in the landscape of abdominal wall repair [5]. Subsequently, different improvements have been proposed to minimize the drawbacks of laparoscopic IPOM, like recurrence, bulging and postoperative pain, together with the problems related to the intraperitoneal mesh placement such as mesh adhesions, fistulation, and migration [6]. During the last years, new minimally invasive approaches have been introduced to overcome the limitations of laparoscopic IPOM, often combining laparoscopic and endoscopic approaches [7]. Most of them have their own characteristics in terms of surgical technique and approach, but some are quite comparable even if called differently [8]. Currently there are no definitive data that may guide surgeons in the choice of the best technique, each approach has advantages and disadvantages, and from a practical point of view it can be useful to classify the different procedures based on the type of approach to the abdominal wall, which can be anterior or posterior to the rectus muscle. This systematic review aims to classify and summarize the characteristics and outcomes of the new laparoendoscopic extraperitoneal techniques for the repair of ventral hernias and DR described in the last 10 years. This study does not aim to establish the superiority of one approach over another, but to understand how the characteristics of each technique can present some advantages based on the indication, always considering the preferences, the experience, and the personal skills of the surgeon.

## Materials and methods

A literature search was performed by two reviewers in December 2023 including articles from January 2013, 01 to December 2023, 15 and using the following databases: Scopus, MEDLINE/Pubmed, Cochrane Library, and Web of Science. A manual search from references to other articles was also performed. The following Medical Subjects Heading (MeSH) terms were used: ((minimally invasive surgical procedures [MeSH Terms]) OR (laparoscopic surgery[MeSH Terms]) OR (endoscopic surgical procedure[MeSH Terms])) AND ((abdominal hernia[MeSH Terms]) OR (hernia, ventral[MeSH Terms]) OR (diastasis[MeSH Terms])). “IPOM”, “robotic”, “IPOM+”, “IPOM plus”, “hiatal”, “groin”, “pediatric”, and “TAPP” terms, together with case reports, editorials, letters to the editor, articles not in English and full text not available were excluded. Additional research for existing reviews, meta-analyses and guidelines was also performed. When more articles were published by the same institution, the most recent was selected. Studies about the extended-view Totally Extra-Peritoneal (eTEP) technique were also excluded because several articles have been published in the last years, including a systematic review and metanalysis in 2022, so it needs to be analyzed in a dedicated study. Studies about techniques with main intraperitoneal working space or transperitoneal approach were also excluded. This review was registered in *protocols.io* with the registration DOI: 10.17504/protocols.io.eq2lyjk3wlx9/v2. The Preferred Reporting Items for Systematic Reviews and Meta-Analyses (PRISMA) [9] and Methodological Index for NOn-Randomized Studies guidelines (MINORS) [10] scoring systems were used for the quality assessment of the studies included in this review. Each manuscript had a MINORS score assessed by two authors (Table 1). Articles were selected according to the inclusion and exclusion criteria based on titles, abstracts, and full-text screening process. After selection, the following information was extracted from each article and reported in a database: bibliographic reference, publication year, technique name (when available), number of patients, sex, age (mean), surgical indications, defect size (mean/area), indications to surgery, surgical time (mean), surgical access type, mesh type, mesh location, post-operative stay, follow-up time, complications (surgical site complications, seroma, other complications), recurrences. The techniques were selected according to the surgical access site (anterior or posterior to the rectus sheath), the access type (laparoendoscopic, single incision laparoscopic, mini or less open), the main space used to repair the defect (subcutaneous or retromuscular), and the mesh place (onlay, sublay-retromuscular or sublay-preperitoneal), and classified as anterior or posterior approaches.

**Table 1:** Articles on the surgical techniques and quality scoring by publication date

Reference	Name	Year	Country	Area	MINORS
Schwarz et al. [15]	EMILOS	2016	Germany	Europe	6/16
Kockerling et al. [47]	ELAR	2017	Germany	Europe	5/16
Kohler et al. [48]	MILAR	2018	Austria	Europe	9/16
Barchi et al. [49]	SVAWD	2018	Brazil	South America	11/16
Li et al. [34]	TES	2018	China	Asia	8/16
Claus et al. [42]	SCOLA	2018	Brazil	South America	11/16
Reinpold et al. [36]	MILOS	2018	Germany	Europe	22/24
Fiori et al. [43]	TESAR	2019	Italy	Europe	8/16
Muas et al. [50]	REPA	2019	Argentina	South America	10/16
Dong et al. [51]	SCOLA	2020	USA	North America	11/16
Kler et al. [52]	TESLAR	2020	UK	Europe	8/16
Li et al. [37]	TEA	2020	China	Asia	9/16
Gandhi et al. [53]	EPAR	2020	India	Asia	9/16
Manetti et al. [28]	No Name	2020	Italy	Europe	7/16
Carrara et al. [29]	THT	2020	Italy	Europe	12/16
Fiori et al. [44]	TESAR	2020	Italy	Europe	19/24
Moga et al. [38]	e-Rives	2021	Romania	Europe	7/16
Li et al. [35]	eTPA	2021	China	Asia	9/16
Cuccomarino et al. [54]	REPA	2021	Italy	Europe	9/16
Makam et al. [55]	SCOM	2022	India	Asia	8/16
Bellido-Luque et al. [16]	FESSA	2022	Spain	Europe	19/24
Shinde et al. [56]	SCOLA modified	2022	India	Asia	7/16
Wang et al. [33]	SIL-TES	2022	China	Asia	19/24
De Carvalho et al. [31]	EMILOS	2023	Brazil	South America	4/16
Nakabayashi et al. [32]	E-MILOP	2023	Japan	Asia	10/16
Signorini et al. [57]	REPA	2023	Argentina	South America	11/16
Ngo et al. [17]	Bilayer technique	2023	France	Europe	12/16

## Results

**Fig. 1:**
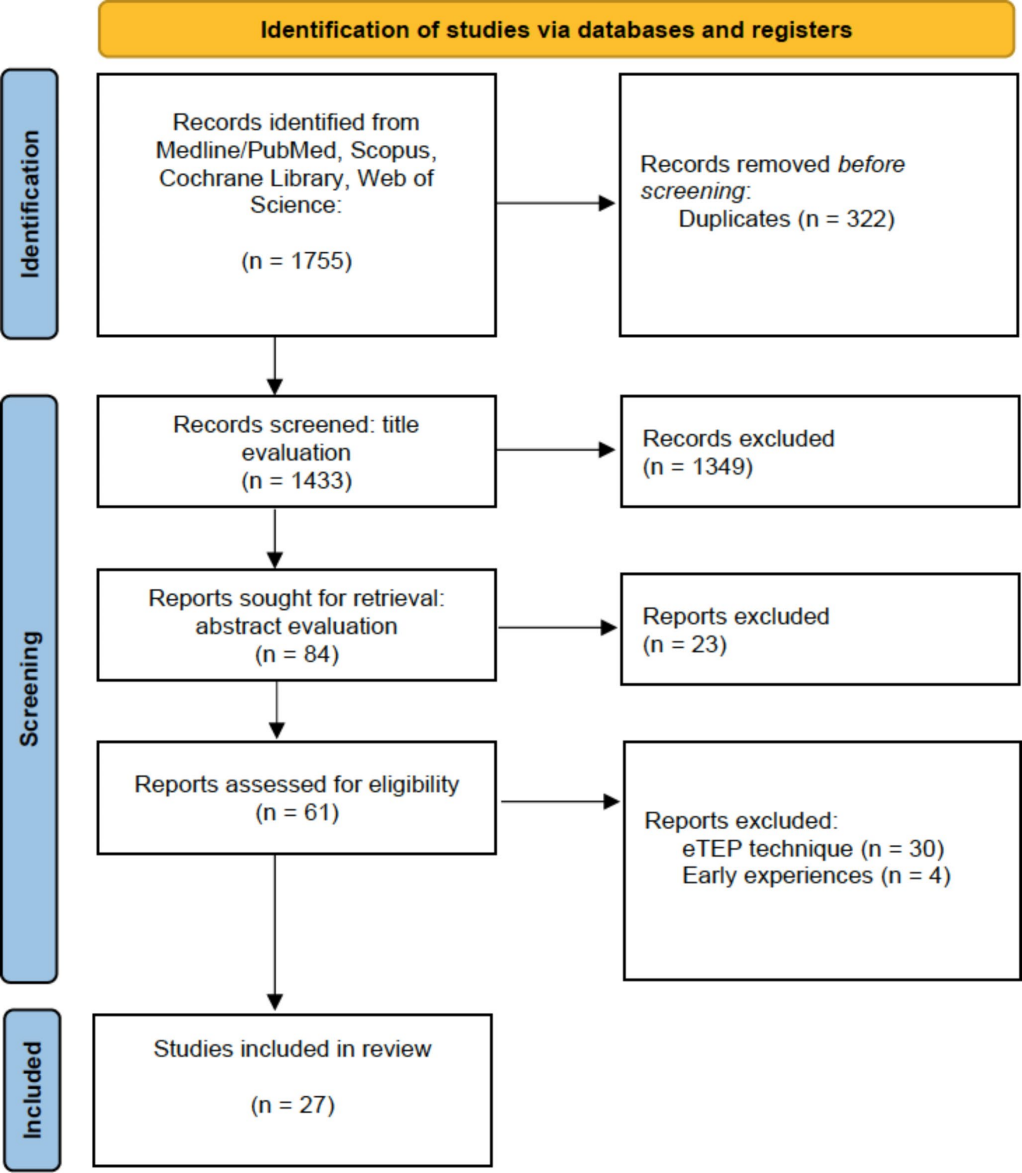
PRISMA 2020 flow diagram for the selection of studies

The literature search retrieved 1755 results, of which 322 were duplicates and excluded from the analysis. After the title evaluation, 1349 other articles were excluded. The abstracts of the remaining 84 articles were analyzed and other 23 studies were excluded because they were not related to the purposes of our review. Of the remaining 61 articles, 30 were about the eTEP technique and 4 were early experiences [11–14], so they were excluded according to the criteria of our review. Finally, 27 articles have been selected for our study. The 2020 PRISMA flowchart with each step of the selection process is presented in Fig. 1. Qualitative assessment of the studies using the MINORS score system showed that none of the studies in this review reached the maximum global score of 16 (non-comparative studies) and 24 (comparative studies). The maximum score was 12/16 in 2 non-comparative studies and 22/24 in one comparative study. The studies were published from 2016 to 2023 and the institutions were world widely distributed, with one study from North America, 5 from South America, 13 from Europe and 8 from Asia (Table 1). The 27 studies included a total number of 1874 patients (range 8–615), with 650 male and 1010 female patients. Two studies [15, 16] with 25 and 28 patients included, did not specify the sex. The mean of patients in the studies was 70.4, but after excluding 4 studies with more than 100 patients the mean dropped down to 31.9 patients. The mean age ranged from 37.8 to 60.2 years (Table 2). The primary indication was ventral hernia (primary and/or incisional) for 19 techniques and DR for 8 techniques. The hernia width was specified in 18 studies, ranging from 15 to 80 mm, and the Inter-Recti Distance (IRD) in 9 studies ranging from 26 to 60 mm. According to the classification used for the techniques, 21 used laparoendoscopic, 5 mini or less open and 1 single-incision laparoscopic access type, whereas the access site was anterior in 16 cases and posterior in 11 cases (Table 3). The mean operative time ranged from 60 to 285 min. The mesh was positioned onlay in 13 cases and sublay in 13 cases, with only one study using no mesh. In 21 studies a polypropylene mesh was used. When declared, the post-operative stay ranged from 0.7 to 4.5 days. In one institution 68 patients were operated on in day-case surgery [17]. The most frequent complication was seroma, ranging from 0.8 to 81%, followed by skin complications (leak, ischemia, necrosis) from 0.8 to 6.4%. Recurrences were reported in 10 studies, ranging from 1.6 to 12.5%, with a mean follow-up from 1 to 24 months (Table 4). On Table 5a summary of anterior and posterior approaches results and on Table 6a brief description of all the techniques according to the classification used in this study are reported.

**Table 2:** Main outcomes of the surgical techniques

Reference	Number of patients	M	F	Age (years, mean)	Hernia width (mm, mean)	IRD (mm)	Operative time (mins, mean)	PO stay (days, mean)
Schwarz et al. [15]	25	nd	nd	53.4	35.5 cm2 (area)	nd	155.0	3.2
Kockerling et al. [47]	140	90	50	54.7	59.0	nd	116.0	4.5
Kohler et al. [48]	20	3	17	41.0	15.0	nd	79.0	4.1
Barchi et al. [49]	21	12	9	47.5	74.0	32.0	112.0	1.0
Li et al. [34]	26	7	19	48.6	33.0	nd	106.0	2.8
Claus et al. [42]	48	20	28	44.3	23.0	41.0	93.5	nd
Reinpold et al. [36]	615	322	293	60.2	75.6 cm 2 (area)	nd	103.0	nd
Fiori et al. [43]	12	5	7	37.8	46.0	nd	148.0	2.6
Muas et al. [50]	201	3	47	38.0	nd	nd	98.0	1.3
Dong et al. [51]	16	2	14	45.7	19.0	nd	146.0	nd
Kler et al. [52]	21	8	13	53.0	nd	nd	nd	nd
Li et al. [37]	28	10	18	50.2	23.0	nd	102.3	1.9
Gandhi et al. [53]	38	14	24	42.0	38.0	nd	85.0	nd
Manetti et al. [28]	74	9	65	46.3	nd	47.0	90.0	nd
Carrara et al. [29]	110	8	102	43.0	16.0	49.0	82.4	2.1
Fiori et al. [44]	26	2	24	43.0	nd	55.0	195.0	3.0
Moga et al. [38]	16	10	6	51.0	20–50	40–60	285.0	2.5
Li et al. [35]	20	11	9	52.2	22.0	nd	105.3	1.8
Cuccomarino et al. [54]	124	6	118	42.0	nd	nd	129.0	nd
Makam et al. [55]	20	7	13	47.0	80.0	nd	117.0	nd
Bellido-Luque et al. [16]	28	nd	nd	52.4	37.0	57.0	70.2	1.4
Shinde et al. [56]	30	20	10	42.3	21.0	nd	110.0	nd
Wang et al. [33]	50	18	22	57.0	14.6 cm2 (area)	nd	145.5	4.3
De Carvalho et al. [31]	8	2	6	46.6	43.0	nd	210.0	1.8
Nakabayashi et al. [32]	26	18	8	53.1	10a50	nd	97.5	1.9
Signorini et al. [57]	54	29	25	50.7	nd	26.0	104.2	0.7
Ngo et al. [17]	77	14	63	40.0	15.0	60.0	60.0	68 pts in Day Case

M: male patients; F: female patients; IRD: inter-recti distance; PO: postoperative

**Table 3:** Main technical features of the surgical techniques

Reference	Primary indication	Other indications	Access site	Access type	Main Working space	Mesh type	Mesh site
Schwarz et al. [15]	Ventral hernia (primary)	DR	Posterior	Mini or less open	Retromuscular	Polypropylene, PVDF	Sublay
Kockerling et al. [47]	DR	Ventral hernia (primary)	Anterior	LAPEND	Subcutaneous	Polypropylene	Onlay
Kohler et al. [48]	DR	Ventral hernia (primary)	Anterior	Mini or less open	Subcutaneous	Byosinthetic absorbable	Onlay
Barchi et al. [49]	Ventral hernia (primary/incisional)	DR	Anterior	LAPEND	Subcutaneous	Polypropylene	Onlay
Li et al. [34]	Ventral hernia (primary/incisional)	nd	Posterior	LAPEND	Retromuscular	PVDF	Sublay
Claus et al. [42]	DR	Ventral hernia (primary/incisional)	Anterior	LAPEND	Subcutaneous	Polypropylene	Onlay
Reinpold et al. [36]	Ventral hernia (primary)	nd	Posterior	LAPEND	Retromuscular	Polypropylene, PVDF	Sublay
Fiori et al. [43]	Ventral hernia (primary/incisional)	DR	Anterior	LAPEND	Subcutaneous	Polypropylene	Sublay
Muas et al. [50]	DR	Ventral hernia (primary)	Anterior	LAPEND	Subcutaneous	Polypropylene	Onlay
Dong et al. [51]	DR	Ventral hernia (primary/incisional)	Anterior	LAPEND	Subcutaneous	Polypropylene, self-fixating	Onlay
Kler et al. [52]	Ventral hernia (primary/incisional)	DR	Anterior	LAPEND	Subcutaneous	Composite, biological	Onlay
Li et al. [37]	Ventral hernia (primary)	DR	Posterior	LAPEND	Retromuscular	PVDF	Sublay-preperitoneal
Gandhi et al. [53]	Ventral hernia (primary/incisional)	DR	Anterior	LAPEND	Subcutaneous	Polypropylene	Onlay
Manetti et al. [28]	DR	Ventral hernia (primary)	Posterior*	LAPEND	Retromuscular	Polypropylene	Sublay
Carrara et al. [29]	Ventral hernia (primary)	DR	Posterior*	LAPEND	Retromuscular	Syntethic, byosinthetic	sublay
Fiori et al. [44]	DR	Ventral hernia (primary)	Anterior	LAPEND	Subcutaneous	Polypropylene	Sublay
Moga et al. [38]	Ventral hernia (primary)	DR	Posterior	LAPEND	Retromuscular	Polypropylene	Sublay
Li et al. [35]	Ventral hernia (primary/incisional)	nd	Posterior	LAPEND	Preperitoneal	Polypropylene	Sublay-preperitoneal
Cuccomarino et al. [54]	DR	Ventral hernia (primary)	Anterior	LAPEND	Subcutaneous	Polypropylene	Onlay
Makam et al. [55]	Ventral hernia (primary)	DR	Anterior	LAPEND	Subcutaneous	Polypropylene	Onlay
Bellido-Luque et al. [16]	Ventral hernia (primary/incisional)	DR	Anterior	LAPEND	Subcutaneous	Polypropylene	Onlay
Shinde et al. [56]	Ventral hernia (primary)	DR	Anterior	LAPEND	Subcutaneous	Polypropylene	Onlay
Wang et al. [33]	Ventral hernia (primary)	nd	Posterior	SILS	Retromuscular	Polypropylene	Sublay
De Carvalho et al. [31]	Ventral hernia (primary/incisional)	nd	Posterior	Mini or less open	Retromuscular	Polypropylene	Sublay
Nakabayashi et al. [32]	Ventral hernia (primary/incisional)	nd	Posterior	Mini or less open	Retromuscular	Polypropylene	Sublay
Signorini et al. [57]	Ventral hernia (primary/incisional)	DR	Anterior	LAPEND	Subcutaneous	Polypropylene	Onlay
Ngo et al. [17]	Ventral hernia (primary/incisional)	DR	Anterior	Mini or less open	Subcutaneous	No	No

LAPEND: laparoendoscopic access; SILS: single incision laparoscopic access; *stapler techniques

**Table 4:** Main complications of the surgical techniques

Reference	Wound complications	Seroma	Other surgical complications	Recurrence	Mean follow-up (months)
Schwarz et al. [15]	4.0%	nd	SSI	0.0%	nd
Kockerling et al. [47]	6.4%	4.8%	bleeding	0.0%	1.0
Kohler et al. [48]	nd	5.0%	nd	5.0%	5.0
Barchi et al. [49]	nd	4.7%	SSI	0.0%	14.0
Li et al. [34]	nd	3.8%	no	0.0%	9.2
Claus et al. [42]	nd	27.0%	SSI	2.1%	8.0
Reinpold et al. [36]	nd	0.8%	Bleeding	1.6%	12.0
Fiori et al. [43]	nd	8.3%	nd	0.0%	nd
Muas et al. [50]	nd	9.7%	nd	0.0%	12.0
Dong et al. [51]	nd	18.8%	SSI	12.5%	2.0
Kler et al. [52]	nd	81.0%	SSI	4.8%	nd
Li et al. [37]	3.6%	7.1%	no	0.0%	18.0
Gandhi et al. [53]	2.6%	5.2%	nd	0.0%	24.0
Manetti et al. [28]	nd	nd	Bleeding	2.7%	6.0
Carrara et al. [29]	3.6%	0.9%	Bleeding. SSI	0.0%	14.4
Fiori et al. [44]	nd	nd	no	0.0%	12.0
Moga et al. [38]	nd	nd	no	0.0%	12.0
Li et al. [35]	nd	5.0%	no	0.0%	10.0
Cuccomarino et al. [54]	0.8%	9.7%	SSI	2.4%	18.0
Makam et al. [55]	5.0%	15.0%	SSI	0.0%	14.0
Bellido-Luque et al. [16]	nd	21.0%	no	3.6%	17.3
Shinde et al. [56]	3.3%	6.7%	no	nd	9.0
Wang et al. [33]	nd	nd	SSI	0.0%	12.0
De Carvalho et al. [31]	nd	nd	no	0.0%	13.0
Nakabayashi et al. [32]	nd	3.8%	Bleeding. SSI	0.0%	9.4
Signorini et al. [57]	nd	40.7%	no	1.9%	6.0
Ngo et al. [17]	nd	28.6%	Bleeding	2.6%	19.0

SSI: surgical site infections

**Table 5:** Summary of anterior and posterior approaches results

	Anterior approaches	Posterior approaches
**Total N. patients**	695	998
N. patients range	12–201	8–615
Age means range	37.8–54.7 years	43–60.2 years
Hernia width means range	15–80 mm	16–50 mm
IRD means range	26–60 mm	47–60 mm
Operative time means range	60–195 min	82.4–285 min
Post-operative discharge means range	0.7–4.5 days	1.8–4.3 days
Follow-up means range	1–24 months	6–18 months
Wound complications range	0.8 − 6.4%	3.6 − 4%
Seroma range	4.7 − 81%	0.8 − 7.1%
Other complications	SSI (4 studies) Bleeding (1 study)	SSI (4 studies) Bleeding (4 studies)
Recurrence range	0–12.5%	0–2.7%

**Table 6:** Brief description of the techniques according to the classification proposed in the present study

Approach type	Technique name	Description
Anterior	**ELAR** (Endoscopic-assisted Linea Alba Reconstruction) [47]	Supraumbilical access. Cutting anterior recti sheaths over their entire length and recreating the linea alba by suturing them together to the fascial defect over exposed recti muscles that are covered with synthetic mesh.
	**MILAR** (Minimal Invasive Linea Alba Reconstruction) [48]	Supraumbilical access. Dissection is performed down to the rectus sheaths, which are incised laterally, and the defect medially closed. A fully absorbable synthetic mesh is inserted to replace the rectus sheaths and secured with sutures.
	**SVAWD** (Subcutaneous Videosurgery for Abdominal Wall Defects) [49] **SCOLA** (Subcutaneous Onlay Laparoscopic Approach) [42, 51] **REPA** (Reparacion Endoscopica Pre-Aponeurotica) [54] **EPAR** (Endoscopic Pre-Aponeurotic Repair) [53]	Suprapubic access. Endoscopic preaponeurotic dissection. Reconstruction of the linea alba by preaponeurotic suturing of edges of stretched recti muscles. Placement of an onlay synthetic mesh in the subcutaneous space.
	**TESLAR** (Total Endoscopic‑assisted Linea Alba Reconstruction) [52]	Like the previous ones, but using composite or biological mesh
	**FESSA** (Full Endoscopic Suprapubic Subcutaneous Access) [16]	Suprapubic access. Endoscopic preaponeurotic dissection. An incision is made on the anterior rectus sheath bilaterally exposing the bellies of both rectus muscles. The two resected medial segments of the anterior layer of the rectus sheath are sutured together in midline. Onlay mesh is positioned and sutured to the lateral incision margins of the anterior rectus sheath opening.
	**SCOM** ([55]laparoscopic Subcutaneous Onlay Mesh)	Lateral access. Endoscopic preaponeurotic dissection. Reconstruction of the linea alba by preaponeurotic suturing of edges of stretched recti muscles. Placement of an onlay synthetic mesh in the subcutaneous space.
	**SCOLA modified** (Subcutaneous Onlay Laparoscopic Approach modified) [56]	Same as SCOLA, but with more limited lateral dissection and a modified access port, used for both camera and energy device.
	**Bilayer technique** [17]	Two steps: open periumbilical incision to suture the hernia and approximate the rectus muscles, followed by endoscopic phase where further suturing of anterior rectus sheath is done to reinforce the repair.
	**TESAR** (Totally Endoscopic Sublay Anterior repair) [43]	Suprapubic access. Endoscopic preaponeurotic dissection. Incision of the medial margins of anterior rectus sheaths. Retromuscular syntethic mesh placement and closing of the anterior rectus sheaths.
Posterior	**MILOS** (Mini- or Less-open Sublay Operation) [36]	Incision directly above the hernia defect (mini or less open access), dissection of the retromuscular space from the hernia defect peripherally with cutting posterior sheaths of recti muscles.
	**EMILOS** (Endoscopic mini/less open sublay technique) [15, 31]	Like the MILOS technique, but with the use of laparoscopic camera.
	**TES** (Totally Endoscopic Sublay) [34]	Suprapubic access. Dissection of the preperitoneal space and then access to the retromuscular plane through the umbilicus to the xyphoid. Closure of posterior and anterior layers and mesh placement.
	**TEA** (Totally Extraperitoneal Approach) [37]	Suprapubic access. Extensive endoscopic development of the midline extraperitoneal plane and reduction of the hernia sac, the hernia defect is closed and a large mesh is placed in the preperitoneal position.
	**SIL-TES** (Single‑Incision Laparoscopic Total Extra‑peritoneal Sublay) [33]	A port-site single incision is made according to the location of the hernia defect. Retromuscular space is dissected and mesh positioned.
	**eTPA** (Endoscopic top-down Totally Preperitoneal Approach) [35]	The preperitoneal space is entered below the xiphoid, endoscopic development of the plane between the peritoneum and posterior rectus sheath is performed behind the linea alba. The hernia defect is closed and a mesh is placed in the newly created preperitoneal space.
	**e-Rives** (Endoscopic Rives) [38]	Left lateral retrorectus access. Bilateral dissection of retromuscular space. Additional ports: suprapubic and right upper quadrant. Posterior and anterior layers closure. Mesh placement.
	**E-MILOP** (Endoscopic-assisted or endoscopic mini- or less-open preperitoneal) [32]	Incision over the hernia defect and careful entrance into, and development of, the preperitoneal space trans-hernially. A synthetic mesh is placed in the preperitoneal space and the defect closed with sutures.
	A new minimally invasive technique for the repair of diastasis recti [28]	Suprapubic access. The posterior rectus sheath is dissected from the rectus muscle. The posterior sheets of the recti muscles are plicated using an endo-stapler. A mesh is then placed in the retromuscular space on top of the posterior sheet without any fixation.
	**THT** (Trentino Hernia Team) [29]	Lower periumbilical access. The umbilicus is disconnected, and the anterior rectus sheaths are isolated. Access to the retromuscular space through small incision. Accessory trocar is placed in one side to check peritoneal adhesions. A linear stapler is used to tighten the medial margins of the rectus muscles up and down. Then endoscopic phase through a single-port: retromuscular space is dissected and endo-staplers are used to tighten the rectus muscles. Synthetic mesh is placed in the retromuscular space.

## Discussion

In the recent years the pursuit of optimizing hernia repair techniques has given rise to several new minimally invasive approaches, including endoscopic, laparoscopic, and robotic techniques. This integration aims to enhance patient outcomes, reduce postoperative complications, and expedite recovery to reduce the limitations of traditional laparoscopic approaches [7, 18, 19].

To our knowledge, this is the first systematic review about the new minimally invasive laparoendoscopic extraperitoneal techniques for the repair of abdominal wall defects.

We have excluded from our analysis intraperitoneal techniques because we believe that the extraperitoneal approach is the major feature that characterizes and differentiates the new approaches from the classic laparoscopic repair techniques (IPOM and IPOM+). We have also excluded transperitoneal techniques, like the ventral TAPP, to limit the study only to total extraperitoneal approaches, and in 2023 a metanalysis on this technique with interesting results has just been published [20]. Moreover, we have excluded studies about robotic hernia repair and the eTEP technique because these approaches need dedicated in-depth analysis due to their wide diffusion in the last years and to remove any possible source of bias in our study, because data about these approaches are more extensive and homogenous than those included in this systematic review. As regards the eTEP, we found a systematic review and meta-analysis published in 2022 including 13 studies and several more articles have been published during 2022–2024 [21]. In the last Update of Guidelines for laparoscopic treatment of ventral and incisional abdominal wall hernias, published in 2019 by the International Endohernia Society (IEHS) [7], there is a chapter dedicated to the new techniques for minimal invasive extraperitoneal mesh repair of abdominal wall hernias and rectus diastasis. The authors, after a review of the published techniques from 2003 to 2018, try to introduce a classification according to the surgical access, location of mesh, modality of defect closure, reconstruction of the abdominal wall and if simultaneous minimally invasive posterior component separation/transversus abdominis release (PCS/TAR) is possible.

In our review, we have identified 27 studies including surgical approaches with different names and some technical differences (Table 1). As already pointed out in a previous review article about endoscopic subcutaneous onlay repair techniques, the same surgical technique has been often published under different names during the last years while describing the same surgical concept with minor technical differences [8]. In effect many ways can be proposed to classify the wide range of new techniques, and in our study we tried to select them according to the access type (laparoendoscopic, single incision laparoscopic, mini or less open), the main working camera (subcutaneous, retromuscular or intraperitoneal) and to the space used to place the mesh (onlay, sublay or intraperitoneal). It is difficult to standardize treatment algorithms because there are too many similarities and, at the same time, some differences between the various techniques. Personal skills may play a significant role in the choice of the technique, and it would be useful to define the added value points of one technique compared to another, or at least of some techniques compared to others, for specific indications, to be able to define appropriate treatment strategies. For the discussion purposes of this study, we have chosen one of the proposed classifications, according to the access site to the abdominal wall, which can be posterior (intra- or extraperitoneal) and anterior.

### Posterior intraperitoneal and transperitoneal approaches

Minimally invasive abdominal surgery was born the ‘90s with the introduction of IPOM technique [5]. In this case the mesh is positioned as a sort of barrier that covers the defect avoiding the possibility of hernia incarceration, there is no reconstruction of the abdominal wall, which instead occurs in the case of IPOM+, that involves the suture of the defect before mesh placement. Currently, it would seem more correct to indicate the IPOM technique with the acronym IPUM, replacing the O for onlay, a historical legacy of the first acronym, with the U for underlay, because the mesh indicated today as onlay is positioned underneath over the muscles. The LIRA technique, recently proposed by the group of Dr. Salvador Morales-Conde, involves the incision and medial plication of the posterior rectus sheath to reconstruct the closure of the defect and the subsequent positioning of the mesh [22]. Compared to the other techniques, the LIRA reduces tension on the suture line, determines the adhesion of the mesh directly in contact with the muscle, and seems to guarantee greater grip of the same with less possibility of detachment.

However, these approaches have an increased risk of adhesions, bowel injuries and mesh-related complications, such as infection, migration, or seroma formation, due to the intraperitoneal mesh positioning and fixation [23, 24]. Moreover, increased postoperative pain [25], and higher reoperation rates have also been described [26].

Ventral TAPP is a transperitoneal approach proposed to overcome the limitations of intraperitoneal techniques. In fact, in a recent metanalysis, it was associated with considerable benefits when compared to IPOM: ventral TAPP was less painful and presented reduced average cost and decreased SSI. However, ventral TAPP and IPOM did not show any difference in terms of intraoperative complications, recurrence rate and chronic pain [20].

So, to overcome the limitations of these techniques, extraperitoneal approaches have been proposed during the last years.

### Posterior extraperitoneal approach

The main advantage of the posterior extraperitoneal approach is to perform a sublay repair working, in most cases, in the same space where the mesh is then placed, without entering the abdominal cavity. As already mentioned, the most diffused sublay endoscopic repair technique proposed in the last years is the eTEP [21]. As specified above, due to its wide diffusion and the relatively large number of published articles, this approach was excluded from our review, and we focused the attention on all the other approaches proposed to perform a retromuscular repair by posterior access. In fact, the systematic review and metanalysis published in 2022 including 13 studies, concluded that eTEP is a promising and safe procedure [21] and several more studies have been published during the last years.

Nevertheless, we cannot discuss about the posterior approaches without comparing them to the eTEP, that today can be considered the main reference for this group. This technique was first published in 2012, based on Daes’ experience in the inguinal hernia and involves direct access to the retromuscular space without entering the abdominal cavity [27]. In the systematic review and metanalysis by Aliseda et al., this approach presented good results in terms of surgical site infection (0%), seroma (5%), major complications (1%), intraoperative complications (2%), conversion rate (1%), mean hospital length of stay (1.77 days) and recurrence rate (1%) [21]. In our study 11 techniques were included in this group, in which bleeding has been the most reported complication, maybe related to the dissection in the retromuscular space. Seroma and SSI were not significantly reported, with seroma rates ranging from 0.8 to 7.1%, that is comparable to the rates reported for the eTEP. Recurrence rates ranged from 0 to 2.7%, but follow-up is reported only from 6 months to a maximum of 18 months. The mean hospital stay ranged from 1.8 to 4.3 days, superior to the value reported for the eTEP. The authors emphasize how some complications like injury to the linea alba, retromuscular hematoma or injury to the neurovascular bundles could, theoretically, increase morbidity and reoperation rates especially at the beginning of the learning curve. So, they conclude that this procedure needs to be performed in the hands of well-trained hernia surgeons [21].

In this group there are also two techniques that use staplers for the section-suture of the fascia [28, 29]. These are mainly extraperitoneal, but the peritoneal cavity is always evaluated for the possible risk of visceral injuries during the use of the stapler, especially in the case of visceral adhesions. Both were indicated for the treatment of ventral hernias and DR, with good results in terms of technical difficulty (operative time 82.4–90 min), complications (seroma rate 0.9%, wound complications rate 3.6% for THT) and recurrence rate (0-2.7%). The promising good results of this approach face with some problems that regard the tightness of the posterior plane during the THT technique, due to the tension caused by the medial plication that occurs during the mechanic section-suture, especially in the case of large defects. The Trentino Hernia Team compensates for this with a release of the posterior sheath of the rectus medial to the neurovascular bundles to reduce tension on the posterior fascia, or by performing a partial TAR. This measure eliminates tension on the rear surface, lowering sealing problems [30]. However, the size of the defects currently presented in the literature does not indicate these techniques for large secondary defects. In fact, the two studies included in this review reported a mean inter-recti distance of 47–49 mm and a mean hernia width of only 16 mm (Table 2).

The other posterior techniques presented a mini or less open access in 2 approaches [15, 31, 32], a single incision access in one case [33] and laparoendoscopic approach in 5 cases [34–38]. They were all comparable in terms of operation time (97.5–285 min) and they were indicated for small hernias (width 22–50 mm). Only in the e-Rives the IRD was reported (40–60 mm) [38]. The MILOS study presented the largest cohort of patients of all the studies included in this systematic review, with 615 patients and a follow-up of 12 months, the authors reported 1.6% of recurrence rate [36]. Recurrences were registered only in the studies by Manetti et al. [28] and Reinpold et al. [36], 2.7% and 1.6%, respectively. All the other studies did not registered recurrences, but this is obviously related to the short follow-up (9.2–18 months) and to the small cohort of patients included in the studies. As regards the complications, seromas ranged from 0.8 to 7.1%, and wound complications were reported only in two studies (3.6% and 4%) [15, 37]. Bleeding was registered in 4 studies, maybe due to the dissection in the retromuscular space [28, 29, 32, 36].

In summary, all the posterior extraperitoneal approaches present a specific feature: there is one main working space (retromuscular) with a low risk of seroma because there is no preaponeurotic detachment and low risk of intra-abdominal injury due to the almost totally extraperitoneal nature of the approach. They give optimal functional results with no drain needed usually. However, on the other hand, they may present, depending on the type and size of the defect as well as the morphology of the patient, little vertical bulging at the skin level, reported and described sometimes as temporary, or the presence of a residual hernial sac included in the repair suture as a possible site of persistent seroma [39]. Furthermore, the learning curve is quite long [40]. In our review, among the 11 studies presenting a posterior extraperitoneal approach, one placed the mesh preperitoneal [35] and 10 in the retromuscular space and they were mainly indicated for the repair of ventral hernias. The learning curve was never investigated and there is no report about the morphological outcomes.

### Anterior approach

According to the access site, 16 techniques used the anterior approach with subcutaneous space as the main work camera. Most of them were just analyzed and discussed in a previous review focused on endoscopic subcutaneous onlay repair, in which the authors underline the similarities among the different names proposed for the same surgical technique and propose to unify them under one term, Endoscopic Onlay Repair (ENDOR) [8]. Since the article published by Bellido-Luque in 2015 [11], the preaponeurotic plane has been increasingly considered as a space of possible use for the treatment of midline defects. After the publication of Bellido-Luque, other authors published the same approach almost simultaneously [41, 42]. Most of the techniques belonging to this group involve the placement of an onlay mesh (Table 3), and they registered low complication rates as well as good results from a functional point of view (wound complications 0.8–6.4%, other surgical complications reported: SSI in 6 studies, bleeding in 2 studies). However, they are mainly used for the repair of DR with small umbilical hernias. The inter-recti distance reported in the studies included in this review ranged between 32 and 60 mm and the mean hernia width between 15 and 80 mm. In two anterior approaches the mesh is not placed onlay. In the first, the bilayer technique by Philippe Ngo [17], there is no mention of mesh. The use of mesh in abdominal wall repair has become a standard practice in modern surgical procedures because it significantly reduces the risk of recurrence allowing for a tension-free repair and leading to better outcomes and reduced postoperative pain for patients. Therefore, no-mesh repair should not be considered nowadays, especially in the case of complex abdominal wall defects like midline hernias and DR with IRD greater than 50 mm. The second is the TESAR technique, published by our group in 2019 for the repair of ventral and incisional hernias [43] and in 2021 for DR and umbilical hernias [44]. This approach is the only technique to date that provides anterior access with retromuscular mesh repair. We believe that this procedure has some advantages and that it is indicated not only for the repair of defects such as DR and umbilical hernias, like most anterior approach techniques, but that it can be considered among the possible options of choice in patients with secondary defects at high risk of intraperitoneal adhesions. On the other hand, the onlay repair carries some other controversial aspects, like the presumed increased risk of complications. As reported in the present study, among these approaches the risk of seroma ranged from 4.7 to 81% and there was also an increased risk of SSI reported in the different experiences. Moreover, there is an increased risk of recurrence, compared to the sublay repair (Table 4). The studies included in this review reported a recurrence rate of 1.9–12.5%, and 6 studies reported no recurrences, but with limited follow-up (1–24 months). In fact, the retromuscular space is often considered as the best positioning plane for a mesh in the literature, considering it safer in terms of surgical site infections (SSI) and recurrence [45, 46]. Nevertheless, the main advantage of the anterior approaches is the safety, with a very low risk of visceral injury, and no need to work against abdominal pressure and with the instruments in reverse, like in the posterior approaches. It is also possible to remove all the hernia sac, giving an optimal morphological outcome, especially in thin patients.

### General considerations and limits

Overall, the studies included in this review, are from Europe in 14 cases, followed by Asia (9 studies) and America (6 studies), showing a worldwide tendency to develop new approaches for the repair of abdominal wall defects. The real limitation of these studies is the poor population, in most cases the number of patients included does not overcome 50 cases, in fact after excluding the only 4 studies with more than 100 patients, the mean number of patients included was about 30, and in one study the number of patients included was only 8. Only the study of Reinpold et al. included a large number of patients (*n* = 615) [36]. Most of them are case series or retrospective studies with poor follow-up that, when reported, ranges from 1 to 24 months. So, the results should be taken with caution, especially those about the recurrence rates, that in most cases are reported as null.

Posterior approaches are quite similar to the eTEP, which can be considered as the main reference in this field. Anterior approaches with onlay mesh are in most cases the same technique called in differently, as already highlighted in a previous article [8]. TESAR is, to date, the only anterior approach performing a sublay repair [43, 44].

As reported in Table 5, the posterior approaches were investigated in a relatively larger group of patients (998 vs. 695, posterior vs. anterior, respectively), but they were used to repair smaller hernias (16–50 mm vs. 15–80 mm, posterior vs. anterior, respectively). Operative times were longer for the posterior approaches (82.4–285 min) in comparison to the anterior techniques (60–195 min), and this can be related to the possible higher difficulty in performing these approaches that may present a steep learning curve. Seroma rate was higher in the anterior approaches (4.7 − 81% vs. 0.8 − 7.1%, anterior vs. posterior, respectively) and this is linked to the wide subcutaneous dissection performed with these techniques. On the other hand, the posterior approaches presented a higher possibility of bleeding, maybe due to the dissection in the retromuscular space.

The limitations of this review are related to the different populations of patients included in the selected studies, the heterogeneity of the studies with different inclusion criteria, poor follow-up, and scarce outcome data.

## Conclusion

This systematic review confirms the presence of different new minimally invasive techniques for the repair of abdominal wall defects that have been proposed in recent years. Anterior approaches seem easier to perform with good functional and morphological outcomes, but they present high seroma rates. Posterior techniques have a steep learning curve and higher risk of bleeding, but they involve dissection in only one space with very low risk of seroma. All of them have the advantage of performing extraperitoneal abdominal wall repair without the risks of entering the abdominal cavity, like the classic intraperitoneal and transperitoneal approaches. Further studies, with more extensive data about follow-up on homogenous and wider patients’ groups, are necessary to define treatment algorithms to correlate specific indications for specific techniques.

## Data Availability

No datasets were generated or analysed during the current study.
